# Human Microglia Transplanted in Rat Focal Ischemia Brain Induce Neuroprotection and Behavioral Improvement

**DOI:** 10.1371/journal.pone.0011746

**Published:** 2010-07-23

**Authors:** Dashdemberel Narantuya, Atsushi Nagai, Abdullah Md. Sheikh, Junichi Masuda, Shotai Kobayashi, Shuhei Yamaguchi, Seung U. Kim

**Affiliations:** 1 Department of Neurology, Shimane University School of Medicine, Izumo, Japan; 2 Department of Laboratory Medicine, Shimane University School of Medicine, Izumo, Japan; 3 Shimane University School of Medicine, Shimane University Hospital, Izumo, Japan; 4 Division of Neurology, Department of Medicine, UBC Hospital, University of British Columbia, Vancouver, Canada; 5 Medical Research Institute, Chung-Ang University College of Medicine, Seoul, Korea; University of Nebraska, United States of America

## Abstract

**Background and Purpose:**

Microglia are resident immunocompenent and phagocytic cells of central nervous system (CNS), which produce various cytokines and growth factors in response to injury and thereby regulate disease pathology. The purpose of this study is to investigate the effects of microglial transplantation on focal cerebral ischemia model in rat.

**Methods:**

Transient middle cerebral artery occlusion (MCAO) in rats was induced by the intraluminal filament technique. HMO6 cells, human microglial cell line, were transplanted intravenously at 48 hours after MCAO. Functional tests were performed and the infarct volume was measured at 7 and 14 days after MCAO. Migration and cell survival of transplanted microglial cells and host glial reaction in the brain were studied by immunohistochemistry. Gene expression of neurotrophic factors, cytokines and chemokines in transplanted cells and host rat glial cells was determined by laser capture microdissection (LCM) and quantitative real time-PCR.

**Results:**

HMO6 human microglial cells transplantion group demonstrated significant functional recovery compared with control group. At 7 and 14 days after MCAO, infarct volume was significantly reduced in the HMO group. In the HMO6 group, number of apoptotic cells was time-dependently reduced in the infarct core and penumbra. In addition, number of host rat microglia/macrophages and reactive astrocytes was significantly decreased at 7 and 14 days after MCAO in the penumbra. Gene expression of various neurotrophic factors (GDNF, BDNF, VEGF and BMP7) and anti-inflammatory cytokines (IL4 and IL5) was up-regulated in transplanted HMO6 cells of brain tissue compared with those in culture. The expression of GDNF and VEGF in astrocytes in penumbra was significantly up-regulated in the HMO6 group.

**Conclusions:**

Our results indicate that transplantation of HMO6 human microglial cells reduces ischemic deficits and apoptotic events in stroke animals. The results were mediated by modulation of gliosis and neuroinflammation, and neuroprotection provided by neurotrophic factors of endogenous and transplanted cells-origin.

## Introduction

Microglia are immunocompetent cells of central nervous system (CNS), which are activated in response to injury and diseases, and adopt a phagocytic and cytokine secreting phenotype [1]–[4]. Microglia are activated following in cerebral ischemia and express a variety of proinflammatory cytokines including interleukin -1β (IL-1β), interleukin -6 (IL-6) and tumor necrosis factor -α (TNF-α), which induce in neuroinflammation and neurotoxicity [5]–[8]. It also take part in neuroinflammation through chemokines, such as monocyte chemotactic protein-1 (MCP-1) and macrophage inflammatory protein -1α (MIP -1α), production and recruitment to circulating immune cells [9]–[11].

Conversely, microglia is known to produce neurotrophic factors such as glial cell line-derived neurotrophic factor (GDNF), brain-derived neurotrophic factor (BDNF), basic fibroblast growth factor (bFGF) and vascular endothelial growth factor (VEGF), potentially provide trophic support to neurons in distress [12]–[14]. These reports suggest that the phenotypic expression of microglia, whether it is to be neuroprotective or neurodegenerative, depends on the cue it receives during a particular disease process.

Regenerative medicine using stem cell-based cell therapy has recently emerged as a therapeutic tool in various disease settings [15]. Various cell types including neural stem cells, mesenchymal stem cells (MSCs) and immortalized stem cell lines have been used for stem cell based therapy in experimental settings, as well as clinical research of stroke in an anticipation that it may improve the disease pathology. Indeed, several resent reports have implicated the beneficial effects of cell-transplantation including neural stem cells, MSCs and genetically engineered stem cell line [16], [17].

Since microglia are able to act neuroprotectively in the CNS in injury or disease, we hypothesized that microglial transplantation could provide neuroprotection of neurons under cerebral ischemic condition. We have previously generated an immortalized cell line of human microglia, HMO6, which carries morphologic and phenotypic expression characteristic of primary human microglia [18], [19].

To test our hypothesis, we intravenously transplanted HMO6 human microglia cells in rat model of transient ischemia, and evaluated histopathological, molecular and functional recovery in these ischemia model animals.

## Materials and Methods

### Cell culture

A human microglial cell line, HMO6, was established by isolating microglia from human fetal telencephalon tissue and immortalizing it using a retroviral vector encoding v-myc [19]. HMO6 cells were cultured in medium consisted of Dulbecco's modified Eagle medium (DMEM; Nissui, Tokyo, Japan) supplemented with 5% fetal bovine serum, L-glutamine, amphotericin B and gentamicin. After 24, 48, 60 and 72 h of growth, cells were exposed briefly to trypsin and then collected by centrifugation. HMO6 cells at final concentration of 3×10^6^ cells per 100 µl DMEM were prepared for transplantation.

### Middle cerebral artery occlusion (MCAO) model

All experimental protocol and procedures were approved by the Ethical Committee of the Shimane University School of Medicine. Adult male Sprague-Dawley rats (n = 60; 7–8 weeks old; CLEA Japan,Tokyo) weighting 250 to 300 g were employed in our experiments. Briefly, rats initially anesthetized with 4% halothane and maintained with 1–2% halothane in 70% N_2_O and 30% O_2_ mixture using a face mask. Transient MCAO was induced using a previously described method of intraluminal vascular occlusion [20]. The right common carotid artery, external carotid artery and internal carotid artery were exposed via a ventral midline incision. A 4-0 monofilament nylon suture (Nescosuture, Tokyo, Japan), with its tip rounded by coated with silicon (Xantopren L blue, Heraeus Kulzer, Hanau, Germany) was inserted from the right external carotid artery into of the internal carotid artery to block the origin of the right middle cerebral artery. At 90 minutes after MCAO, occluded animals were re-anesthetized with halothane and nylon monofilament was removed, and the end of the external carotid artery was tied. The rats were allowed to recovery from anesthesia and returned to the cages.

### Transplantation of HMO6 cells

In the study, MCAO rats were randomly divided into two experimental groups: Transplantation (n = 30) and control (n = 30) groups. Forty eight hour after MCAO animals were anesthetized and HMO6 cells (3×10^6^ cells/rat) were injected through jugular vein. In control MCAO animals, phosphate-buffered saline (PBS) alone was injected. No immunosuppressant such as cyclosporine A was utilized in the study as well as previous studies since cyclosporine A reduces glial response and exerts neuroprotection in experimental stroke.

### Functional tests

Functional tests were performed at 1, 4, 7, 10 and 14 days after MCAO by the modified neurological severity scores (mNSS). mNSS was used to grade the various aspects of neurological functions, which was adopted from a previous report, with some modification [21]. The modified NSS (mNSS; Table 1) is a composite of motor (i.e., muscle status and abnormal movement), sensory (i.e., visual, tactile, and proprioceptive), beam balance, and reflex tests, in which meticulous sensory examination for vision, touch, and proprioceptive sensation was performed. The total score for the test was 22 points. Increasing score indicates the severity of injury. In a preliminary examination, rats with 12–16 points of mNSS had a stable stroke volume. Hence, we used rats with 12–16 points for further examination.

**Table 1:** Modified NSS.

Motor Tests	Points
Raising rat by the tail	3
1 Flexion of forelimb	
1 Flexion of hindlimb	
1 Head moved >10 to vertical axis within 30 s	
Placing rat on the floor (normal = 0; maximum = 3)	3
0 Normal walk	
1 Inability to walk straight	
2 Circling toward the paretic side	
3 Fall down to the paretic side	
Sensory tests	6
1 Placing test (visual test)	
2 Placing test (tactile test)	
3 Proprioceptive test (deep sensation, pushing the paw against the table edge to stimulate limb muscles)	
Beam balance tests (normal = 0; maximum = 6)	6
0 Balances with steady posture	
1 Grasps side of beam	
2 Hugs the beam and one limb falls down from the beam	
3 Hugs the beam and two limbs fall down from the beam, or spins on beam (>60 s)	
4 Attempts to balance on the beam but falls off (>40 s)	
5 Attempts to balance on the beam but falls off (>20 s)	
6 Falls off: No attempt to balance or hang on to the beam (<20 s)	
Reflexes absent and abnormal movements	4
1 Pinna reflex (head shake when touching the auditory meatus)	
1 Corneal reflex (eye blink when lightly touching the cornea with cotton)	
1 Startle reflex (motor response to a brief noise from snapping a clipboard paper)	
1 Seizures, myoclonus, myodystony	
Maximum points	22

One point is awarded for inability to perform the tasks or for the lack of a tested reflex.

### Triphenyltetrazolium chloride (TTC) staining and evaluation of infarct volume

Rats (8 rats in each group; n = 16) were decapitated under deep anesthesia with an overdose of diethyl ether at 7 and 14 days after MCAO. The brain was immediately removed and sectioned into 2 mm thick slices from the frontal pole to the cerebellum. These sections were stained with 2% 2–3–5 TTC (Sigma) in normal saline at 37°C for 30 minutes, after which sections rinsed 0.9% saline solution for photography. TTC stained sections per animal were photographed using digital high definition microscope (Keyence VH-7000, Osaka, Japan). The six areas of infarction between each adjoining slice were measured using NIH image software, and then infarct volumes (mm^3^) were determined by integrating the appropriate area with the section interval thickness.

### Histological and immunohistochemical assessment

Experimental animals, two groups of 4 each (total n = 32) at 3, 5, 7 and 14 days after MCAO, were re-anesthetized and rat organs were fixed by transcardial perfusion with saline, followed by 4% paraformaldehyde (PFA) in 0.1mol/L phosphate buffer (PB, pH 7.4) and 10% sucrose. The rat tissues (brain, liver, spleen, lung, kidney) were immersed in 20% sucrose for 48 hours and then were embedded in TissueTek OCT compound and frozen on dry ice. The brain tissues were cut equally spaced (thickness 2 mm) coronal blocks, followed by sections sliced into 10 µm in a cryostat. To investigate the histological changes in the brain tissue, haematixiine and eosine (H.E.) staining was performed.

For immunostaining, brain sections were incubated with mouse monoclonal antibody specific to Human nuclei (1∶100; MAB1281, Chemicon, Temecula, CA) to evaluate the migration of transplanted HMO6 cells. Double immunohistochemical staining was performed to identify glial cells accumulation with polyclonal rabbit anti-GFAP antibody (1∶100; Dako, Carpinteria, CA) and monoclonal mouse anti - ED1 antibody (1∶100; Serotec, Oxford, UK). Sections were then incubated for 1 hour at room temperature with secondary antibodies, Texas red- conjugated goat anti-rabbit IgG (1∶100,Chemicon) and FITC-conjugated goat anti-mouse JgG (1∶100; Chemicon). All sections were stained with Hoechst 33258 (10 mg/ml, Sigma) as a nuclei staining. After staining, sections were mounted with ultramount (Dako) and visualized or photographed with fluorescence microscopy system (Nikon, ECLIPSE, E600, Tokyo, Japan). ED1 is specific for mouse/rat microglia/macrophages and not for human.

The terminal deoxynucleotidyl transferase (TdT) - mediated dUTP-biotin nick – end labeling (TUNEL) method was used to assess apoptotic cell death. Brain sections were stained by In Situ Cell Death Detection Kit (Roche Diagnostics, Mannheim, Germany). After quenching endogenous peroxidase activity with 0.3% H_2_ O_2_ in PBS, slides was placed in TdT. A dark brown color indicating DNA breaks developed after incubation with 3, 3′- diaminobenzidine (DAB, Vector Lab, Burlingame, CA).

### Laser capture microdissection (LCM)

Tissue preparation and staining: The animals, two groups of 6 each (total n = 12) at 5 days after MCAO, were deeply anesthetized, decapitated and the brains were quickly removed. The brain sections were snap frozen with liquid nitrogen and stored at −80°C for further use. Ten-µm brain cryosections were prepared, fixed in 75% ethanol for 30s. For detection of HMO6 cells, sections were incubated with anti- human nuclei, for detection of rat glial cells, incubated with, anti- GFAP and anti- ED1 antibody for 1 hour followed by secondary antibody for 30 minutes. During immunostaining, RNase inhibitor was added to all reagents, and aqueous reagents were prepared in with diethylpyrocarbonate (DEPC) treated distilled water.

Immunostaining-positive cells for human nuclei, GFAP or ED1 were respectively isolated from ischemic hemispheres of MCA occluded rats by LCM system (Arcturus, Mountain View, CA), which was equipped with an inverted base microscope system (Olympus, Tokyo, Japan). Microdissections of rat brain sections were performed under 10x objective and target cells were collected from each rat brain on an LCM Cap (CapSure Macro Caps, Arcturus; Mountain View, CA). Following cells collection, total RNA was isolated with the PicoPure RNA isolation kit (Arcturus; CA) according to the manufacturer's instructions.

### Real time PCR

Total RNA of HMO6 cell culture was isolated from confluent HMO6 using RNA STAT reagent (TELTEST) according to the manufacturer's instructions. Briefly, confluent HMO6 cells of 60-mm cell culture dishes were lysed and extracted with chloroform, and the aqueous phase was transferred to a fresh tube. Total RNA was precipitated with 2-propanol, washed once with 70% ethanol, and re-suspended in a suitable volume of DEPC-treated water. The total RNA concentration was determined by measuring the OD values of the samples at 260 nm.

To prepare first strand cDNA, mRNA was reserve transcribed with reverse transcriptase enzyme (ReverTraAce, Toyobo, Osaka, Japan) in a total 20 µl reaction mixture. To analyze mRNA level, real time PCR was performed with SYBR PCR Master Mix (power SYBR green, Applied Biosystems, CA) and an ABI Prism 7000 Sequence Detector system (Applied Biosystems, CA). The list of primer sequences is provided in Table 2. After normalization with GAPDH mRNA, the target gene mRNA level in a sample was quantified by relative quantification method.

**Table 2:** Primers for real time PCR.

Gene	Sense	Antisense	Gene bank ACC number
human GDNF	TTTAGGTACTGCAGCGGCTCTT	TCACTCACCAGCCTTCTATTTCTG	NM000514
human BDNF	ATTACAATCAGATGGGCCACATG	AGGGAGAAAGCAGAAACAAGACA	M61176
human b-FGF	CGACCCTCACATCAAGCTACA	AACGGTTAGCACACACTCCTT	NM002006
human VEGF	GGCCAGCACATAGGAGAGATG	AGGCCCACAGGGATTTTCTT	AF022375
human BMP-7	CGTGGAACATGACAAGGAATTC	CGTGACAGCTTCCCCTTCTG	NM001719
human CNTF	TGTGCGTGCTTGCATGTG	ACCCTGAAGTGGAAGGACGTT	NM000614
human IL-1β	TTACAGTGGCAATGAGGATGA	TGTAGTGGTGGTCGGAGATT	NM000576
human IL-4	AACAGCCTCACAGAGCAGAAGAC	GTGTTCTTGGAGGCAGCAAAG	NM000589
human IL-5	TGGAGCTGCCTACGTGTATGC	GCAGTGCCAAGGTCTCTTTCAC	NM000879
human IL-6	CCTGAGAAAGGAGACATGTAACAAGA	TGGAAGGTTCAGGTTGTTTTCTG	M54894
human IL-8	CTGGCCGTGGCTCTCTTG	TTAGCACTCCTTGGCAAAACTG	NM000584
human IFN-g	GTCCAACGCAAAGCAATACATG	CCTTTTTCGCTTCCCTGTTTTAG	NM000619
hu Fractalkine	CATCACGTGCAGCAAGATGAC	CGCATGATGCCTGGTTCTG	NM134455
human MCP-1	GACCATTGTGGCCAAGGAGAT	TGTCCAGGTGGTCCATGGA	NM002982
hum GAPDH	CCACATCGCTCAGACACCAT	TGACCAGGCGCCCAATA	M33197
rat GDNF	CTCGAAGTAGAAGGCTAACA	AGCGGAATGCTTTCTTAGG	NM017017
rat BDNF	TGTCCGAGGTGGTAGTACTTCATC	CATGCAACCGAAGTATGAAATAACC	NM012842
rat b-FGF	GAGAGAGGAGTTGTGTCCATCAAG	GCAGCCGTCCATCTTCCTT	X61697
rat VEGF	GAGGAAAGGGAAAGGGTCAAAA	CACAGTGAACGCTCCAGGATT	AF062644
rat IL-1β	CACAGCAGCATCTCGACAAGA	CACGGGCAAGACATAGGTAGCT	NM31512
rat IL-4	CAGGGTGCTTCGCAAATTTTAC	ACCGAGAACCCCAGACTTGTT	NM_201270
rat IL-6	TCAACTCCATCTGCCCTTCAG	AAGGCAACTGGCTGGAAGTCT	M26744
rat IL-10	AGAAGCTGAAGACCCTCTGGATAC	GCTCCACTGCCTTGCTTTTATT	L02926
rat TNF-α	CAGCCGATTTGCCACTTCATA	TCCTTAGGGCAAGGGCTCTT	X66539
rat TGF-β	CGTGGAAATCAATGGGATCAG	CAGGAAGGGTCGGTTCATGT	NM021578
rat IL-8	GAAGATAGATTGCACCGA	CATAGCCTCTCACACATTTC	
rat MCP-1	CCAGAAACCAGCCAACTCTCA	AAGCGTGACAGAGACCTGCAT	NM031530
rat MIP-1α	CATTCCTGCCACCTGCAAAT	CAAGTGAAGAGTCCCTGGATGTG	NM013025
rat iNOS	AAGAACTCGGGCATACCTTCAG	GTCATGAGCAAAGGCACAGAAC	NM012611
rat GAPDH	CAGCCTCGTCTCATAGACAAGATG	AAGGCAGCCCTGGTAACCA	AF106860

### Statistical Analysis

Data were expressed as mean ± standard error of the mean (S.E.M). Experimental data were analyzed by analysis of variance and means with ANOVA followed by Fisher's PLSD test for the difference between means. A values of P<0.05 and P<0.01 were considered statistically significant.

## Results

### Neurological functional recovery

**Figure 1:**
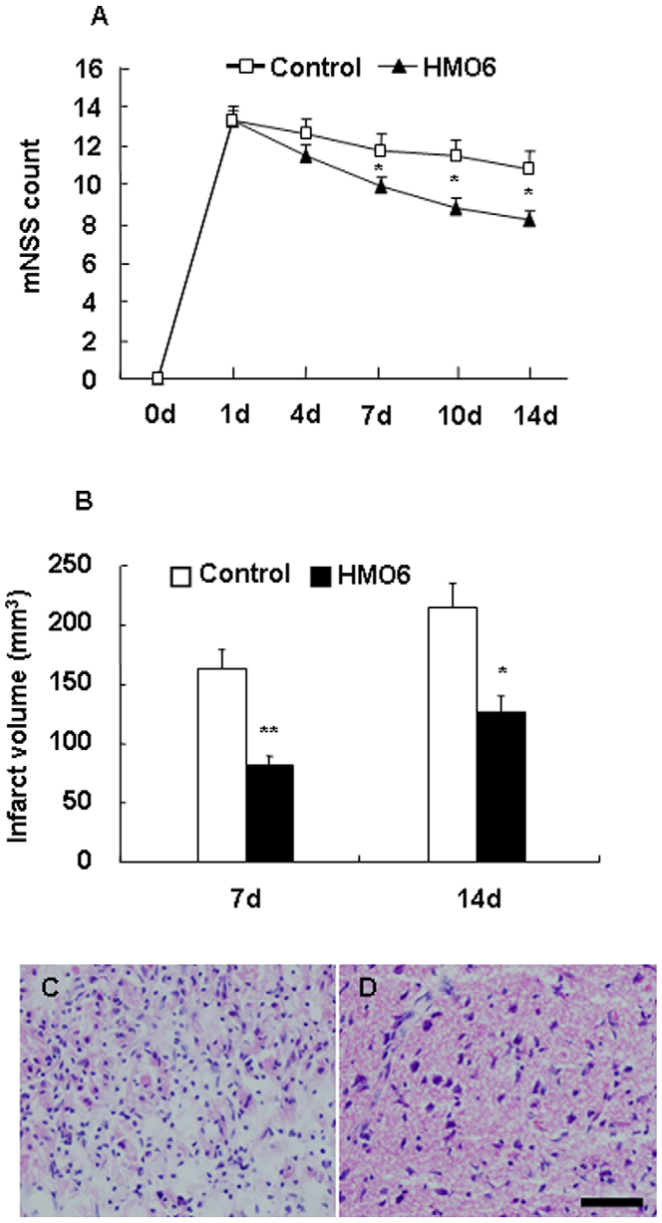
Effect of HMO6 transplantation on neurological function, infarct volume and tissue damage. (A) Neurological function of the experimental groups after MCAO was determined by the modified Neurological Severity Scores (mNSS). The mNSS summarizes the results of motor, sensory, reflex, and balance tests. Transplantation of HMO6 human microglial cells enhanced neurological functional recovery at 7, 10 and 14 days after MCAO. (B) Infarct volume was calculated at 7 and 14 days after MCAO in the HMO6 transplantation and control groups based on TTC staining and NIH image software. Data of mNSS and infarct volume is expressed as the mean ± SEM. *P<0.05, ** P<0.01, as compared with the control group. H.E. staining of the penumbra lesion in a control rat (C) and a transplanted rat (D) at 7 days after MCAO. Bar  = 100 µm.

We investigated whether transplanted human microglial cell, HMO6 improves neurological deficits after MCAO in rat. The mNSS scores at 1 and 4 days were not significant different between control group and HMO6 transplantation group. However, neurological functional recovery was significantly found at 7, 10 and 14 days after MCAO in the HMO6 transplantation group compared with the control group (Figure 1A).

### Measurement of Infarct volume and histological changes

Histological analysis of ischemic lesions indicated that at 7 days after MCAO the mean infarct volume was 163±17 mm^3^ in the control group and 91±16 mm^3^ in the HMO6 transplanted group (44% reduction in infarct volume), and at 14 days after MCAO the infarct volume was 214±29 mm^3^ in the control and 127±20 mm^3^ in the HMO6 group (41% reduction in infarct volume) (Figure 1B). There were significant reductions in infarct volumes following transplantation of HMO6 human microglia in MCAO animals at 7 and 14 days after MCAO. Tissue damage with disarrangement and vacuoles and cell infiltration in infarct core and penumbra were higher in control groups compared to those in transplanted groups during the time course (Figure 1C and 1D).

### Apoptotic cells

**Figure 2:**
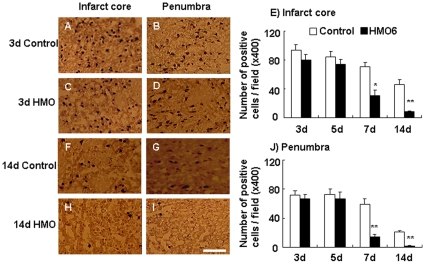
Effect of HMO6 transplantation on apoptosis. TUNEL positive cells were shown in the HMO6 transplantation (C, D, H and I) and control group (A, B, F and G) at 3 (A-D) and 14 (F-I) days after MCAO. Number of apoptotic cells was determined in the infarct core (E) and the penumbra (J). Number of apoptotic cells is expressed as the mean ± SEM. Bar  = 20 µm, *P<0.05, ** P<0.01, as compared with the control group.

Number of apoptotic cells was counted in the ischemic core and the penumbra at 3, 5, 7 and 14 days after MCAO in brain sections processed for TUNEL staining. Number of apoptotic cells at 3 and 5 days in the core (Figure 2E) and the ischemic penumbra (Figure 2J) were not significant different between the HMO6 transplantation group and the control group.

At 7 days after MCAO, the number of apoptotic cells within the ischemic core was 70.9±5.9 cells/field in the control group and 30.4±7.6 cells/field in the HMO6 transplantation group respectively (41% reduction in number of apoptotic cells) (Figure 2E). At 14 days after MCAO, the number of apoptotic cells within the ischemic core was 45.7±6.6 in the control (Figure 2F) and 8.2±1.4 in the HMO6 group respectively (82% reduction in number of apoptotic cells) (Figure 2H and 2E).

At 7 days after MCAO, the number of apoptotic cells within the penumbra was 59.1±7.9 cells/field in the control group and 14.7±3.1 cells/field in the HMO6 transplantation group respectively (75% reduction in number of apoptotic cells) (Figure 2J). At 14 days after MCAO, the number of apoptotic cells within the penumbra was 20.9±2.1 in the control (Figure 2G) and 1.9±0.4 in the HMO6 group respectively (91% reduction in number of apoptotic cells) (Figure 2I and 2J).

### Migration of HMO6 human microglial cells

**Figure 3:**
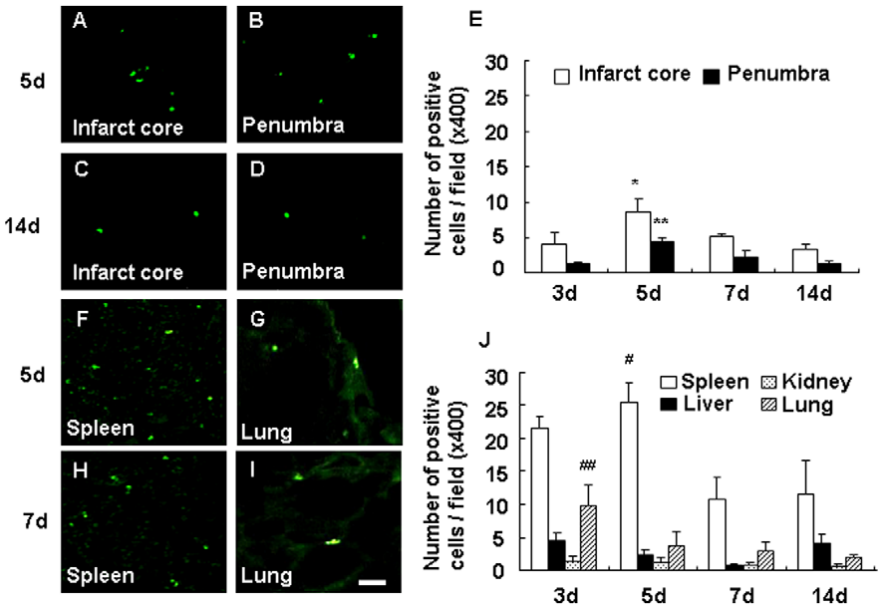
Distribution of human nuclei positive cells after HMO6 cell transplantation. Human nuclei (HuN, FITC) positive cells were detected by fluorescence microscope at 5 (A and B) and 14 (C and D) days after MCAO in the infarct core (A and C) and the penumbra (B and D) of the brain. Histogram (E) shows the number of HuN-positive cells in infarct core (empty bars) and penumbra (solid bars), counted at X400 magnification. HuN-positive cells in internal organs were shown at 5 (F and G) and 7 (H and I) days in the spleen (F and H) and lung (G and I). Histogram (J) shows the number of HuN-positive cells in spleen, liver, kidney and lung, counted at X400 magnification in the time course after MCAO. Number of HuN-positive cells is expressed as the mean ± SEM. Bar  = 20 µm, * P<0.05 vs. infarct core at 3 and 14 days, ** P<0.01. vs. penumbra at 3, 7 and 14 days. ^#^ P<0.05 vs. spleen at 7 and 14 days, ^##^ P<0.05 vs. lung at 7 and 14 days.

At 3, 5, 7 and 14 days after MCAO, migration of HMO6 cells into brain was examined by human nuclei (HuN) staining. In the brain, HuN-positive HMO6 cells were found in survive infarct core and penumbra in the ipsilateral hemisphere, while in the contralateral hemisphere HMO6 cells were not found. A peak of HMO6 cell migration was found at 5 days after MCAO (Figure 3A and 3B) as compared with 3, 7 and 14 days (Figure 3C and 3D). Migration declined after the 7 day after MCAO and only a small number of HMO6 cells was detected at 14 days after MCAO (Figure 3E). HMO6 cells were also transplanted in healthy rats to find out whether they enter into normal brains. However, HuN-positive cells were detected only in optic chiasma.

Since HMO6 cells were transplanted intravenously, migration and localization of HMO6 cells in other parts of body were determined. The presence of HuN-positive cells was found in the spleen, lung, liver and kidney at 3, 5, 7 and 14 days. HMO6 cell migration in the spleen was significantly higher at 5 days (Figure 3F and 3J) as compared with 7 and 14 days (Figure 3H and 3J). Number of HuN-positive cells was significantly higher at 3 days in the lung as compared with 7 days and the number of cells was much lower oncompared with 7 and 14 days (Figure 3J).

### Effect of HMO6 transplantation on glial cell accumulation

**Figure 4:**
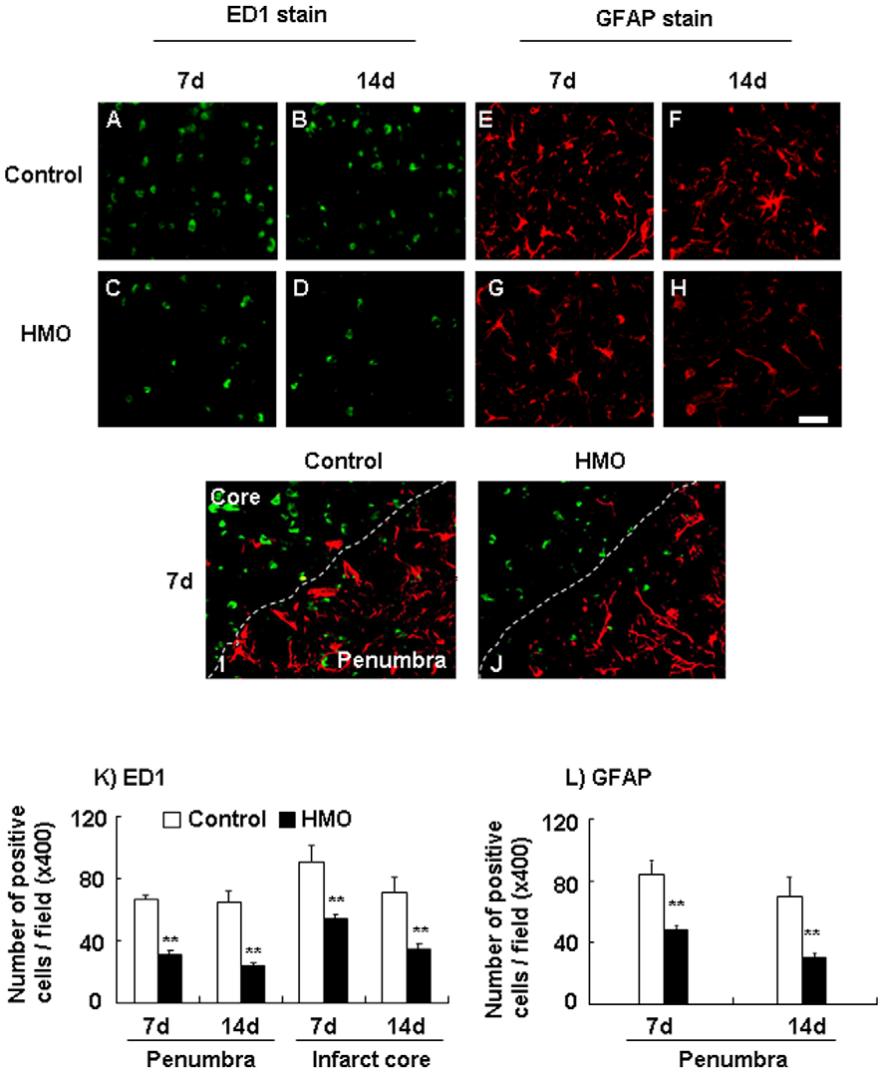
ED1 and GFAP positive cells in the penumbra. Immunohistochemical reactions to ED1 (A-D: FITC) and GFAP (E-H: Texas red) were shown at 7 (A, C, E and G) and 14 (B, D, F and H) days after MCAO. Double staining with anti-ED1 and anti-GFAP antibodies was detected in the penumbra of the HMO6 transplantation group (J) and control group (I) at 7 days after MCAO. Histograms show the density of ED1-positive microglia/macrophages (K) and GFAP-positive astrocytes (L) in the HMO6 transplantation and control groups of 7 and 14 days after MCAO. Number of positive cells is expressed as the mean ± SEM. Bar  = 20 µm, ** P<0.01 compared to the control group.

Effect of HMO6 transplantation on glial reaction in the ischemic core and the penumbra region was investigated with ED1 and GFAP immunostaining. ED1-positive cells, representing host rat microglia/macrophages, were found in the ischemic core to penumbra of the ipsilateral hemisphere. Number of ED1-positive cells was smaller in the penumbra as compared with the ischemic core (Figure 4I and 4J). In the ischemic core, number of round-shaped ED1 positive cells at 7 and 14 days after MCAO was significantly decreased in the HMO6 transplantation group as compared with control group. At 7 days after MCAO, the number of ED1-positive cells was 90.4±6.4 cells/field in the control group (Figure 4A) and 55.2±1.7 cells/field in the HMO6 transplantation group respectively (39% reduction in number of apoptotic cells) (Figure 4C). At 14 days after MCAO, the number of ED1-positive cells was 70.9±5.9 cells/field in the control (Figure 4B) and 35.1±1.9 cells/field in the HMO6 group respectively (50% reduction in number of apoptotic cells) (Figure 4D).

In the penumbra at 7 days after MCAO, number of ED1-positive cells was 68.2±4.4 cells/field in the control group (Figure 4K) and 29.1±4.7 cells/field in the HMO6 transplantation group respectively (57% reduction in number of ED1 cells) (Figure 4K). At 14 days after MCAO, the number of ED1-positive cells was 59.1±2.5 cells/field in the control (Figure 4K) and 23.7±1.1 cells/field in the HMO6 group respectively (60% reduction in number of ED1 cells) (Figure 4K).

At 7 days after MCAO, number of GFAP-positive cells was 84.3±4.5 cells/field in the control group (Figure 4E and 4L) and 48.2±1.5 cells/field in the HMO6 transplantation group respectively (43% reduction in number of GFAP cells) (Figure 4G and 4L). At 14 days after MCAO, the number of GFAP-positive cells was 70.1±7.1 cells/field in the control (Figure 4F and 4L) and 30.2±1.7 cells/field in the HMO group respectively (57% reduction in number of GFAP cells) (Figure 4H and 4L).

### Gene expression of neurotrophic factors, cytokines and chemokines in HMO6 cells in culture and brain tissue

**Figure 5:**
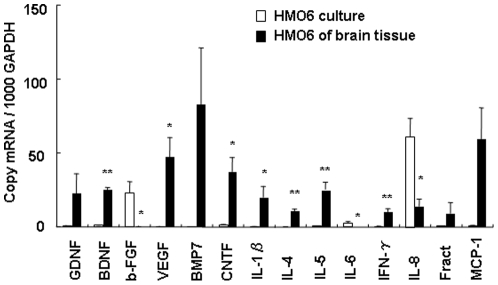
Gene expression in HMO6 cell *in vitro* and *in vivo*. Gene expression of neurotrophic factors, cytokines and chemokines in the HMO6 cell of culture (open bars) and brain tissue (solid bars) was analyzed by quantitative real-time PCR. Glyceraldehyde-3- phosphate dehydrogenase (GAPDH) was used as a reaction standard. Gene expression was determined in culture HMO6 cells under normal condition. HMO6 cells of brain tissue were isolated from infarct core and penumbra at 5 days after MCAO by laser capture microdissection (LCM). Values represent mean ± SEM from three experiments.

Gene expression of neurotrophic factors, cytokines and chemokines was determined in HMO6 cells in culture and the brain by real time-PCR (Figure 5). The results showed that expression of b-FGF and IL-8 in HMO6 cells were high, while expression of GDNF, BDNF, CNTF, IL-5, IL-6, IFN-γ, fractalkine and MCP-1 was low and expression of VEGF, BMP-7, IL-1β, IL-4 in HMO6 cells was not detected and under un-stimulated *in vitro* culture conditions.

To study the actual gene expression in transplanted HMO6 cells in the brain, HMO6 cells from brain tissue at 5 days after MCAO were isolated by the use of with LCM and gene expression of neurotrophic factors, cytokines and chemokines was measured by real time-PCR. Expressions of GDNF, BDNF, VEGF, BMP-7, CNTF, IL-1β, IL-4, IL-5, IFN-γ, fractalkine and MCP-1 were up-regulated in HMO6 cells of brain tissue as compared with those in culture. On the other hand, expressions of b-FGF, IL-6 and IL-8 were down-regulated in HMO6 cells of brain tissue compared with HMO6 cell culture.

### Gene expression of neurotrophic factors, cytokines and chemokines in rat microglia and astrocytes of brain tissue

To determine the effect of the HMO6 transplantation on host brain microglia and astrocyte, gene expression of neurotrophic factors, cytokines and chemokines was investigated in rat glial cells of the penumbra from brain tissue with the LCM at 5 days after MCAO by real-time PCR.

**Figure 6:**
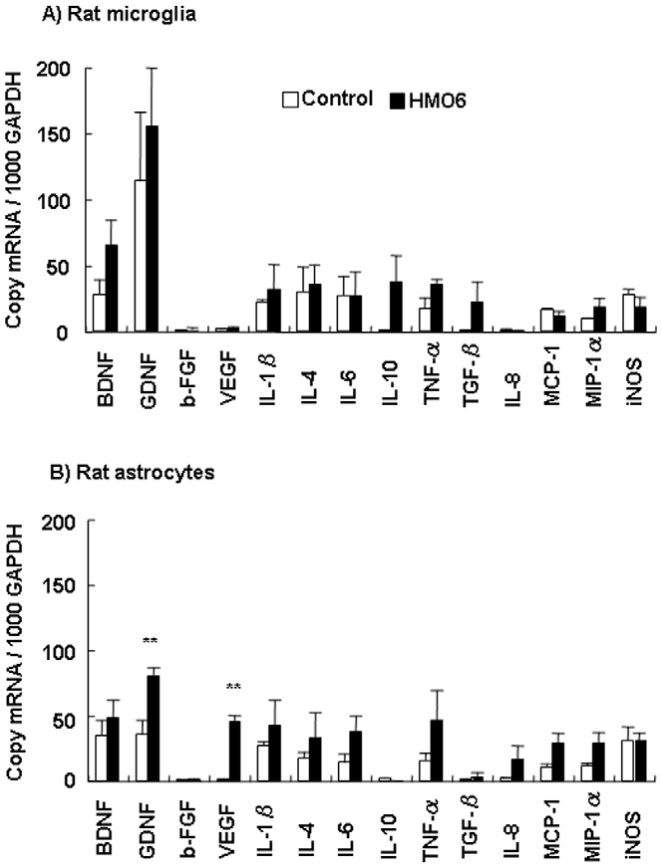
Gene expression in rat glial cells. Gene expression of neurotrophic factors, cytokines and chemokines in rat ED1-positive microglia/macrophages (A) and GFAP-positive astrocytes (B) was analyzed by quantitative real-time PCR. ED1- and GFAP-positive cells were isolated from penumbra of the HMO6 transplantation and control groups at 5 days after MCAO by laser capture microdissection. Glyceraldehyde-3- phosphate dehydrogenase (GAPDH) was used as a reaction standard. Expressional levels of each factor were calculated in the transplantation and control groups. Values represent mean ± SEM from three experiments. **P<0.01 compared to the control group.

There were no significant differences between transplanted group and control group in expression of neurotrophic factors, cytokines and chemokines in rat microglia/macrophages (Figure 6A). The expressions of GDNF and VEGF in astrocytes of penumbra were significantly up-regulated in the transplantation group as compared with the control group. Expressions of BDNF, IL-1β, IL-4, IL-6, TNF-α, TGF-β, IL-8, MCP-1 and MIP-1α were higher in astrocytes of transplanted brain than those in astrocytes of control brain, though not significantly different (Figure 6B).

## Discussion

The major findings of the present study are that intravenous transplantation of human microglial cells significantly improved the ischemia-induced functional and morphological changes in the ipsilateral hemisphere after t-MCAO.

Previous studies have reported that intravenous transplantation of neural stem cells (NSCs), MSCs and human umbilical cord blood cells (HUCBC) significantly improves neurological deficits after experimental stroke [15], [17], [21], [22], which raised the possibility of cell therapy for ischemic stroke. Most of stem cells/precursor cells are believed to enter into brain through disrupted blood-brain barrier and migrate to the ipsilateral hemisphere, especially to the ischemic border zone expressed as penumbra [15], [23], which is also supported by our data that HMO6 cells transplanted in healthy rats did not enter the brains except optic chiasma. HMO6 human microglial cells transplanted in MCAO rats also were found in penumbra, however, the cell density was higher along infarct core. As expression of cytokines and chemokines are upregulated in the penumbra, transplanted microglial cells carrying respective receptors are thought to be chemo-attracted and recruited and migrate along the infarct core [9], [24]. Tissue damage in the infarct core and penumbra was not induced by HMO cell transplantation and migration, rather an improvement of histological tissue architecture was observed. On the contrary, migrated HMO6 cells almost disappeared from the brain 14 d after MCAO. Fate of HMO6 cells localized in the lesion should be determined in the further study.

In the rodent MCAO studies, TUNEL-positive cells in the ischemic lesion peaked at about one week after MCAO and were continuously seen for several weeks [25], [26]. Previous reports have indicated that systemically transplanted stem cells reach the ischemic lesion and have a neuroprotective effect to reduce apoptotic neuronal cell death in host neurons and that reduction of stroke volume began after the event. Neuroprotective effect to reduce the number of apoptotic cells was found at 7days after MCAO in the present study and then, infarct volume of both PBS control and HMO transplantation groups continued to increase up to two weeks as seen in previous studies [27], [28]. The reason remains elusive, but various factors including apoptotic signal pathway, necrosis and repair process might affect it. Transplantation of bone marrow stromal cells and GDNF-gene modified NSCs reduced the number of TUNEL-positive cells, caspase-3-positive cells and also neuronal degeneration from the acute phase to several months after MCAO [25], [29], [30], which was consistent with microglial transplantation in this study.

Brain injury following focal cerebral ischemia triggers activation of microglia and astrogliosis and induction of released free radicals, inflammatory cytokines and proteases [31], [32], [33], [34].The activated microglia releasing reactive oxygen species, nitric oxide, chemokines and inflammatory cytokines[35], [36] leading to accumulation and activation of more blood born and resident immune cells. Moreover, these microglial inflammatory products are highly neurotoxic. The study in cerebral ischemic model demonstrated that the application of immunosuppressant (FK506) reduced delayed neuronal death by glial/microglial inhibition [37]. In the present study, significantly fewer number of host rat ED1-positive microglia/macrophage was detected in the infarct core and penumbra in HMO6 human microglia-transplanted brains. It appears that transplantation of HMO6 human microglia in the ischemic brain induces reduction in host glial activation that leads to abated neurotoxicity. In addition, transplanted HMO6 human microglia showed increase expression of several neurotrophic factors and Th2 type cytokines, which might be involved in glial inhibition and also protection of host neurons. In the transplanted brain, the number of GFAP-positive astrocytes in the penumbra was lower indicating curtailed astrogliosis in the lesion site. Recently, it is proposed that astrocytes contribute to delayed neuronal death by release of cytotoxic cytokines after ischemia [38], [39]. The present results of abated astrogliosis and the reduction in the number of TUNEL-positive cells in the penumbra are in good agreement with these studies.

In the HMO6-transplanted group, reduced infarct volume and reduction in the number of glial cells in penumbra were observed from 7 days after MCAO and onward. It appears that the transplanted HMO6 human microglial cells attenuate glial reaction of host glial cells, which leads to reduce infarct volume, by increased expression of neurotrophic factors, cytokines and chemokines. It is interesting to note that several neurotrophic factors such as GDNF, BDNF, BMP-7, CNTF and VEGF were upregulated in the transplanted HMO6 human microglial cells as compared to HMO6 cells in culture. Increased expression of these trophic factors in transplanted HMO6 cells might have resulted from inducing signals at the microenvironment of the ischemic brain. In the present results, anti-inflammatory cytokines such as IL-4 and IL-5 were also increased although IL-1β, a proinflammatory cytokine and MCP-1, a chemokine, were also increased. These results indicate that the gene expression in the transplanted HMO6 human microglial cells was dramatically changed in the microenvironment of ischemic brain lesion site.

Previous reports demonstrated that transplantation of gene-modified human MSCs overepressing GDNF, BDNF, or VEGF after cerebral ischemia reduce infarct volume and ameliorate neurological deficits [40], [41], [42]. High levels of GDNF, BDNF and VEGF were found in transplanted HMO6 cells in ischemic lesion site and responsible for restration of behavior and neuroprotection. BMP-7, a trophic factor expressed by HMO6 cells, is a member of transforming growth factor-β superfamily, the receptors are found in neuron and astrocytes [43] and known to promote neuroregenerative effect in stroke rats [44]. Since BMP7 was highly expressed in HMO6 cells in penumbra, it is one of the neuroprotective factors secreted by the transplanted HMO6 human microglial cells in the ischemic brain.

Previous studies have demonstrated that the brain transplantation of human neural stem cells overexpressing VEGF or GDNF in cerebral hemorrhage stroke animal models promoted functional recovery and neuronal protection [45], [46]. The transplantation of bone marrow stromal cells also promoted expression of VEGF in host brain, and enhance angiogenesis and functional repair in the ischemia brain [47]. The present study showed that the transplantation of HMO6 cells in the ischemic brain induced a significant up-regulation in expression of GDNF and VEGF in ischemic penumbra. The upregulated GDNF and VEGF in the rat brain might be one of cause for neuronal recovery because those factors can support neuronal survival and attenuate neuronal apoptotic death [48], [49].

In conclusion, the present study demonstrated that the intravenously transplanted human microglia migrate to ischemic area, and provide neuroprotection via producing neurotrophic factors and cytokines and reducing endogenous glial response. Selective accumulation of transplanted microglia in lesion core and penumbral area indicate that that microglia could be an effective vehicle for transfer of therapeutic genes for gene therapy in neurological diseases including cerebral ischemic disease.
